# Maternal Dietary *Forsythia suspensa* Extract Supplementation Induces Changes in Offspring Antioxidant Status, Inflammatory Responses, Intestinal Development, and Microbial Community of Sows

**DOI:** 10.3389/fvets.2022.926822

**Published:** 2022-07-15

**Authors:** Shenfei Long, Qianqian Wang, Tengfei He, Jiayu Ma, Jian Wang, Sujie Liu, Hongliang Wang, Li Liu, Xiangshu Piao

**Affiliations:** ^1^State Key Laboratory of Animal Nutrition, College of Animal Science and Technology, Agricultural University, Beijing, China; ^2^College of Resources and Environmental Sciences, China Agricultural University, Beijing, China; ^3^Tianjin Zhongsheng Feed Co., Ltd., Tianjin, China

**Keywords:** antioxidant capacity, *Forsythia suspensa* extract, inflammatory responses, reproductive performance, sow

## Abstract

This experiment aims to investigate the effect of maternal diet supplemented with *Forsythia suspensa* extract (FSE) on the performance, antioxidant status, inflammatory responses, intestinal development, and microbial community of sows. A total of 24 gestating sows (Landrace × Yorkshire) were assigned to 2 treatments with 12 sows per treatment. From d 107 of gestation to d 21 of lactation, sows were supplemented with a basal diet as control (CON) or an FSE diet (basal diet + 100 mg/kg FSE). Compared with CON, sows fed FSE showed lower (*P* < 0.05) wean-to-estrus interval, body weight loss, and higher (*P* < 0.05) average daily gain of suckling piglet. Sows fed FSE had reduced (*P* < 0.05) serum malondialdehyde (MDA) content and enhanced (*P* < 0.05) catalase and glutathione peroxidase (GSH-Px) contents at farrowing and weaning compared with CON. The suckling piglets of FSE-fed sows had increased (*P* < 0.05) mRNA expressions of nuclear factor erythroid-2 related factor 2, heme oxygenase-1 in the liver, and lower (*P* < 0.05) serum MDA content on d 0, 7, and 14 of lactation. Sows fed FSE had lower (*P* < 0.05) serum tumor necrosis factor-α (TNF-α) and interleukin-8 (IL-8) contents at farrowing and reduced (*P* < 0.05) serum IL-6 and IL-8 contents at weaning compared with CON. Piglets from FSE-fed sows had enhanced (*P* ≤ 0.05) villus height and villus height to crypt depth ratio in the jejunum, and higher (*P* < 0.05) protein expression of Occludin in jejunal mucosa compared with CON. Sows fed FSE tended to have higher (*P* = 0.09) relative abundance of *Lactobacillus* at genus level in feces at weaning compared with CON. Our results showed maternal diet supplemented with FSE in lactating sows could effectively induce improvement of performance, antioxidant status, anti-inflammatory function, intestinal morphology, barrier function, and microbial community.

## Introduction

In modern pig reproduction, the pigs weaned per sow per year (PSY) in China is about 17, while the PSY in modern countries (such as the USA and France) is about 25, thus the low PSY has become a serious problem in China currently. For gestating and lactating sows, the increased mammary development, fetal growth, tissue mobilization, or milk production might cause the overproduction of reactive oxygen species (ROS), which might result in oxidative stress (including the oxidation of lipid and protein) (1). Moreover, from birth to weaning, due to the immature immune system, it is difficult to neutralize the excess free radicals for the piglets, which may lead to the intestinal dysfunction, higher diarrhea incidence, lower nutrient utilization, average daily gain (ADG), and survival rate in suckling piglets (2, 3). Therefore, raising the antioxidant capacity and immune function is the key point to solving the problem of sows and suckling piglets.

For piglets, the maternal antibodies in colostrum and milk are important for alleviating various stresses and improving immune system development (4). Previous studies have demonstrated that maternal dietary seaweed extract or chitosan oligosaccharide supplementation in late gestating and lactating sows could increase the reproductive performance and immune function in sows, as well as enhance the anti-stress ability and immunity of suckling piglet (5, 6). However, the maternal dietary antioxidant supplementation in late gestating and lactating sows on preventing excessive oxidative stress still needed to be re-evaluated (7). Herbs are medicinal and nutritional important in improving the health care of livestock (8). Previous studies in our lab had revealed that *Forsythia suspensa* extract (FSE, one of the herb extracts) was effective in improving anti-inflammatory function and antioxidant status *in vivo* and *in vitro* (9–12). Moreover, FSE could increase intestinal epithelial cell proliferation, nutrient digestibility, and immune function, and therefore enhance the performance in weaned piglets (13). Furthermore, FSE could also alleviate the stresses induced by heat (9), diquat (11), high stocking density (14), corticosterone (15), lipopolysaccharide (16), and transport (17). And the main antioxidant composition of FSE included forsythiaside A, phillygenin, and phillyrin and forythialan A (11). Therefore, we speculated that FSE, a traditional Chinese herb extract, has the potential to solve the oxidative stress problems in sow production.

Studies showed that some functional nutrients or seaweed extracts could enhance the body's immune and antioxidant functions of piglets through maternal transmission (18–21). In our previous studies, we also found that dietary supplementation with FSE could effectively modulate intestinal morphology and microbial community in broilers or weaned pigs (22, 23). While diet supplemented with FSE in late gestating sows could help to increase the nutrient utilization, antioxidant status, and inflammatory responses, and eventually alleviate oxidative stress during farrowing and improve the reproductive performance of sows (24). However, there are still few research focusing on investigating the maternal dietary FSE supplementation on alleviating the physiological stress of lactating sows and enhancing the anti-stress ability, as well as modulating the gut health of offspring by maternal transmission. Based on previous studies in our lab, we hypothesize that maternal FSE supplementation induce changes in antioxidant status, anti-inflammatory functions, intestinal development, and microbial community in their offspring. Therefore, the current research aimed to demonstrate our hypothesis and find the possible maternal transmission of FSE-fed sows on modulating performance, antioxidant status, inflammatory responses, and gut health in suckling piglets, which might be beneficial to supply a way to solve the oxidative stress problem in sow production during late gestation and lactation periods.

## Materials and Methods

The conduction of this experiment was at the China Agricultural University's Feng Ning Swine Research Unit (Chengde, Hebei, China) and the procedure was agreed upon by the China Agricultural University's Institutional Animal Care and Use Committee (Beijing, China, AW70601202-1-1).

### Experimental Products

According to the procedure by the study by Lu et al. (11) in our lab, dried and ground forsythia fruits from Henan province were prepared and extracted with 80% ethanol, sonicated for 1 h, and then filtered. Ethanol was used to extract the residue twice and then rotary vaporization (Buchi, Rotavapor R-124, Flawil, Switzerland) was utilized to dry and combine the filtrates. The major functional ingredients of FSE were forsythiaside A (33.0 mg/kg), phillygenin (33.4 mg/kg), and phillyrin (163.4 mg/kg) and forythialan A (82.6 mg/kg). The additive amount of FSE (100 mg/kg) in the diet of sows also followed the previous studies conducted by Wang et al. (9) and Zhang et al. (14).

### Experimental Design, Animals, and Diets

Based on initial body weight, parity, and backfat thickness, 24 gestating sows (Landrace × Yorkshire, average body weight of 234 ± 6.81 kg, average parity of 3.38 ± 0.61) were assigned to 2 treatments with 12 sows per treatment. From d 107 of gestation to d 21 of lactation (weaning day), sows were provided with a basal diet as control (CON) or an FSE diet (basal diet + 100 mg/kg FSE). In the basal diet, the nutrient composition met or exceeded the requirements recommended by NRC (25) (Table 1).

**Table 1:** Composition and nutrient levels of basal diets (g/kg, as fed basis).

**Item**	
**Ingredients**	
Corn	627.1
Soybean meal	230.0
Wheat bran	80.0
Soy oil	32.0
Dicalcium phosphate	8.0
Limestone	11.5
Salt	3.0
L-Lysine HCl, 78%	0.8
L-Valine, 99%	2.6
Vitamin-Mineral premix^a^	5.0
**Nutrients levels**	
Digestible energy, kcal/kg^b^	3,389
Crude protein^c^	170.0
Digestible phosphorus^b^	2.9
Calcium^c^	6.9
Lysine^c^	9.1
Threonine^c^	6.2
Tryptophan^c^	1.8
Methionine + cysteine^c^	5.7
Valine^c^	9.4

a *Premix for per kilogram diet included: vitamin E, 24 IU; vitamin A, 12,000 IU; vitamin D_3_, 2000 IU; vitamin K_3_, 2.0 mg; riboflavin, 6.0 mg; thiamine, 2.0 mg; vitamin B_12_, 24 ng; pyridoxine, 4 mg; pantothenic acid, 20 mg; biotin, 0.4 mg; niacin, 30 mg; folic acid, 3.6 mg; iron, 96 mg; choline chloride, 0.4 mg; zinc, 120 mg; iodine, 0.56 mg; copper, 8.0 mg; selenium, 0.4 mg; manganese, 40 mg*.

b *Calculated values*.

c *Analyzed values*.

### Experimental Management

On d 107 of gestation, after bathing and disinfecting, sows were housed individually in 2.0 × 3.0 m^2^ farrowing crates. The room temperature was maintained at 22.0°C (±1.0°C), the light was supplied from 06:30 a.m. to 4:30 pm and the heat lamp were provided for piglets. Before farrowing, sows were fed at 08:00 a.m. and 4:00 p.m. (2.0 kg/d). After farrowing, sows were fed *ad libitum* at 04:30, 10:30, and 16:30 h every day after farrowing. Within 24 h after farrowing, each litter was adjusted to about 10 piglets and each piglet was treated with tail docking, teeth clipping, and ear notching. Within 72 h after farrowing, these piglets were also provided with subcutaneous iron dextran injections (200 mg per pig). According to the relevant requirements, the immunization procedure was carried out. The room was kept ventilated and was cleaned twice a week.

Feed intake during the lactation period of sows was recorded to test the average daily feed intake (ADFI). The body weight (BW) was weighed by the electronic pound in the range of 500 kg (Beijing Hengda Co. Ltd., Beijing, China), while the backfat thickness (at the last rib head, 6 cm from the midline, P2) was measured by the ultrasonic device (Piglog105; SFK Technology A/S, Herlev, Denmark) on d 107 of gestation, within 2 days after farrowing, and d 21 of lactation (weaning day), respectively (26). The BW loss and backfat loss during lactation of sow were calculated, while the number and BW of piglets in the litter on d 0, 7, 14, and 21 of lactation were recorded to measure the ADG of the piglets. After weaning, the wean-to-estrus interval (WEI) was also recorded.

### Samples Collection

The placenta sample of sows was collected and stored at −20°C. The 10 mL heparinized vacutainer tubes (Becton Dickinson Vacutainer System, Franklin Lakes, NJ) were used to collect blood samples (from the anterior vena cava) of sows (*n* = 12) on d 0 and 21 of lactation, and their offspring (*n* = 12) on d 0, 7, 14, and 21 of lactation. These blood samples were centrifuged at 4°C and 3,000 × *g* for 15 min to get the serum samples (stored at −20°C).

On d 21 of lactation, 12 barrows (closest to the average body weight in each pen, *n* = 6) were selected and injected with pentobarbital sodium for complete anesthesia, then slaughtered for the collection of the duodenum, jejunum, and ileum (about 15 cm) as 1/3 distal part. The jejunal mucosa was carefully scraped using a sterile glass slide, and the left liver samples and the digesta samples in the middle part of the cecum and colon were collected. On d 21 of lactation, the fresh fecal samples of sows (closest to the average body weight in each pen) were also collected. The jejunal mucosa, fecal, and digesta samples were placed in a 2-mL cryotube, frozen in liquid nitrogen immediately, and then stored at −80°C until analysis.

### Chemical Analysis

The feed samples were ground and then passed through a 1-mm sieve for the measurement of crude protein, calcium, lysine, methionine, cysteine, threonine, tryptophan, and valine following the methods of AOAC (27, 28). A spectrophotometer (Leng Guang SFZ1606017568, Shanghai, China) was used to determine the concentrations of catalase (CAT), glutathione peroxidase (GSH-Px), malondialdehyde (MDA), interleukin 6 (IL-6), interleukin 8 (IL-8), interleukin 1β (IL-1β), interleukin 10 (IL-10), and tumor necrosis factor-α (TNF-α) in serum of lactating sows or suckling piglets, as well as the content of GSH-Px, CAT, superoxide dismutase (SOD), total antioxidant capacity (T-AOC), and MDA in the placenta by spectrophotometric methods according to the instructions of the kit's manufacturer (Nanjing Jiancheng Bioengineering Institute, Nanjing, China).

### Analysis of Intestinal Morphology

The histological samples were immediately fixed in 50 mL tubes filled in with 10% neutral buffered formalin for about 48 h, and then washed, excised, dehydrated, and embedded in the paraffin wax. About five transverse sections of these histological tissues were sliced, installed on glass slides, and dyed with eosin and hematoxylin. A calibrated 10-fold eyepiece graticule was used to measure about 15 orientated villi and their adjoining crypts randomly on each slice for the calculation of the average villus height, crypt depth, and their ratio (villus height/crypt depth).

### Protein Extraction and Analysis

Protein extraction and analysis were carried out according to the procedure of Hu et al. (29). The protein from jejunal mucosa was extracted by ProteoJET Total Protein Extraction Kit (Fermentas, Hanover, MD, USA) and its content was determined by BCA Protein Assay Kit (Beyotime, Shanghai, China). The protein was separated by 10% Sodium dodecyl-sulfate polyacrylamide electrophoresis (SDS-PAGE), and then transferred to an activated polyvinylidene difluoride membranes (Bio-Rad Laboratories, Hercules, CA, USA). The membrane was blocked by 5% BSA-TBST for 60 min, washed with TBST for 3 times, and then incubated overnight at 4°C using an appropriate primary antibody. The primary antibodies include Rabbit anti occludin (1:200, Abcam) and Rabbit anti-β-Actin (1:1000, Abcam). After incubation with a goat anti-rabbit IgG secondary antibody for 60 min, the target bands were displayed by Odyssey Infrared Imaging System (LI-COR Biosciences, Lincoln, NE, USA) and analyzed by Quantity One Software (BioRad Laboratories, Hercules, CA, USA). The target protein-to-β-actin protein ratio was used to express the relative abundance of occludin in jejunal mucosa.

### The RNA Extraction and Quantitative Real-Time PCR Analysis

Trizol reagent (CWBIO Biotech Co., Beijing, China) was used to extract the total RNA in the liver samples. A Nanodrop ND-1000 spectrophotometer (Thermo Fisher Scientific, Wilmington, DE) was used to check the integrity and purity of RNA. Following the instructions of the manufacturer, the PrimeScriptRT Reagent Kit (TaKaRa, Dalian, China) was used to conduct the reverse transcription of PCR. In the thermal cycling conditions, the real-time PCR was performed for 3 min (at 95°C), 40 cycles at 95°C for 3 s, and then 60°C for 0.5 min according to the procedure of Zhang et al. (30). The primer sequences of GPx1 (glutathione peroxidase 1), SOD1 (superoxide dismutase 1), HO-1 (heme oxygenase 1), Nrf2 (nuclear factor E2-related factor 2), and glyceraldehyde-3-phosphate dehydrogenase (GAPDH) were listed in Supplementary Table 1. The 2^−ΔΔCT^ method was used to analyze the mRNA expression level relative to GAPDH.

### Analysis of Volatile Fatty Acids in Suckling Piglets

The digesta samples in the cecum and colon of piglets were used for the measurement of VFA contents. About 1.5 g of fresh digesta sample were taken into a centrifuge tube, mixed with 1.5 mL sterile water, and centrifuged at 15,000 × *g* for 15 min (at 4°C). A gas chromatograph sample bottle was used to transfer the supernatant, mixed it with 200 μL meta-phosphoric acid, placed in ice for 30 min, and then centrifuged at 15,000 × *g* for 15 min (at 4°C). The VFA contents in digesta samples were measured by high-performance gas chromatography (HPGC; GC-2014; Shimadzu Corporation) equipped with a hydrogen flame detector and a capillary column (Agilent Technologies, Inc., Wilmington, DE, USA; 30 m long, 0.32 mm diameter, 0.50 μm film) following the procedure mentioned by Long et al. (31).

### Analysis of Gut Microbiota Composition

The DNA was extracted from the fecal samples of sows of pigs using the bacterial DNA Kit (Omega Bio-Tek Inc., Norcross, GA, USA). The DNA concentration and purity were preliminarily evaluated by using the Shimadzu spectrophotometer. The barcoded primers 338F (5′-ACTCCTACGGGAGGCAGCAG-3′) and 806R (5′-GGACTACHVGGGTWTCTAAT-3′) were used to amplify the V3–V4 hypervariable regions of the bacterial 16S rRNA genes. The amplified library was sequenced for paired-end reads of 300 bp on the Illumina Hiseq PE250 platform (Illumina, San Diego, US). Paired-end reads were assembled into longer tags and quality-filtered to remove tags with an average quality score <20, a length of <220 bp, and tags containing > 3 ambiguous bases by PANDAseq. After discarding the singletons, the high-quality tags were clustered into operational classification units (OTUs) using Quantitative Insights Into Microbial Ecology (QIIME) pipeline software (version 1.8.0) (a similarity threshold of 0.97). The operational classification unit (OTU) (97% similarity) was categorized by using UPARSE, and the chimeric sequences were identified and removed by using UCHIME. Based on the analysis of the classification (32), the RDP classifier (at 80% confidence level) to the RDP OTU database (https://rdp.cme.msu.edu/) was performed in this study, while the α-diversity was analyzed by using the QIIME (33, 34). According to Segata et al. (35), the linear discriminant analysis (LDA) effect size (LEfSe) tool was used to analyze the LEfSe. The data presented in the study are deposited in the NCBI repository, accession number PRJNA848636.

### Statistical Analysis

The GLM procedure of SAS (SAS Inst. Inc., Cary, NC, 2008) (36) was used to analyze the data except for the microbiota, while the Univariate procedure of SAS was used to measure the homogeneity and normality of variance. Individual piglet or sow was the experimental unit for all the responses. The R software (version 3.2.2) was used to read normalized OTU for microbiota. The relative abundances of *Lactobacillu*s at the genus level were analyzed by *Student's t-test*. Differences in gut bacterial abundance were analyzed by LDA EffectSize. LEfSe analysis uses the Kruskal–Wallis rank-sum test to detect significantly different abundances and performs LDA scores to estimate the effect size (Threshold: ≥2). The least squares means were presented in all values, a trend for the significance was defined at 0.05 < *P* ≤ 0.10, while the significant difference was defined at *P* ≤ 0.05.

## Results

### Performance of Lactating Sows and Suckling Piglets

As shown in Table 2, sows fed FSE diet showed reduced (*P* < 0.05) body weight loss from farrowing to weaning and WEI compared with CON. Moreover, there was a tendency (*P* = 0.10) of increased ADG of piglets from d 0 to 21 and enhanced (*P* < 0.05) ADG of piglet from d 7 to 21 and d 14 to 21 in sows fed FSE in comparison with CON.

**Table 2:** Effects of *Forsythia suspensa* extract on reproductive performance of sows.

	**CON**	**FSE**	**SEM**	***P*-Value**
Average daily feed intake, kg	5.01	5.11	0.19	0.73
**Sow body weight change, kg**				
Body weight at farrowing	230	228	6.45	0.78
Body weight at weaning	207	211	1.23	0.67
Body weight loss from farrowing to weaning	23.5	16.6	0.65	<0.01
**Sow backfat change, mm**				
Backfat at farrowing	14.4	15.1	1.35	0.71
Backfat at weaning	13.0	13.8	1.29	0.69
Backfat loss from farrowing to weaning	1.38	1.38	0.40	1.00
Wean-to-estrus interval, day	7.63	6.13	0.33	0.01
**Average daily gain of piglet, g/d**				
d 0–d 7	181	185	10.70	0.78
d 0–d 14	209	221	8.01	0.33
d 0–d 21	216	238	8.58	0.10
d 7–d 14	233	261	13.10	0.17
d 7–d 21	231	267	10.70	0.04
d 14–d 21	229	273	11.40	0.03

*SEM means standard error of the mean. n = 12*.

*CON, Control; FSE, Forsythia suspensa extract*.

### Antioxidant Status in Lactating Sows and Suckling Piglets

**Figure 1:**
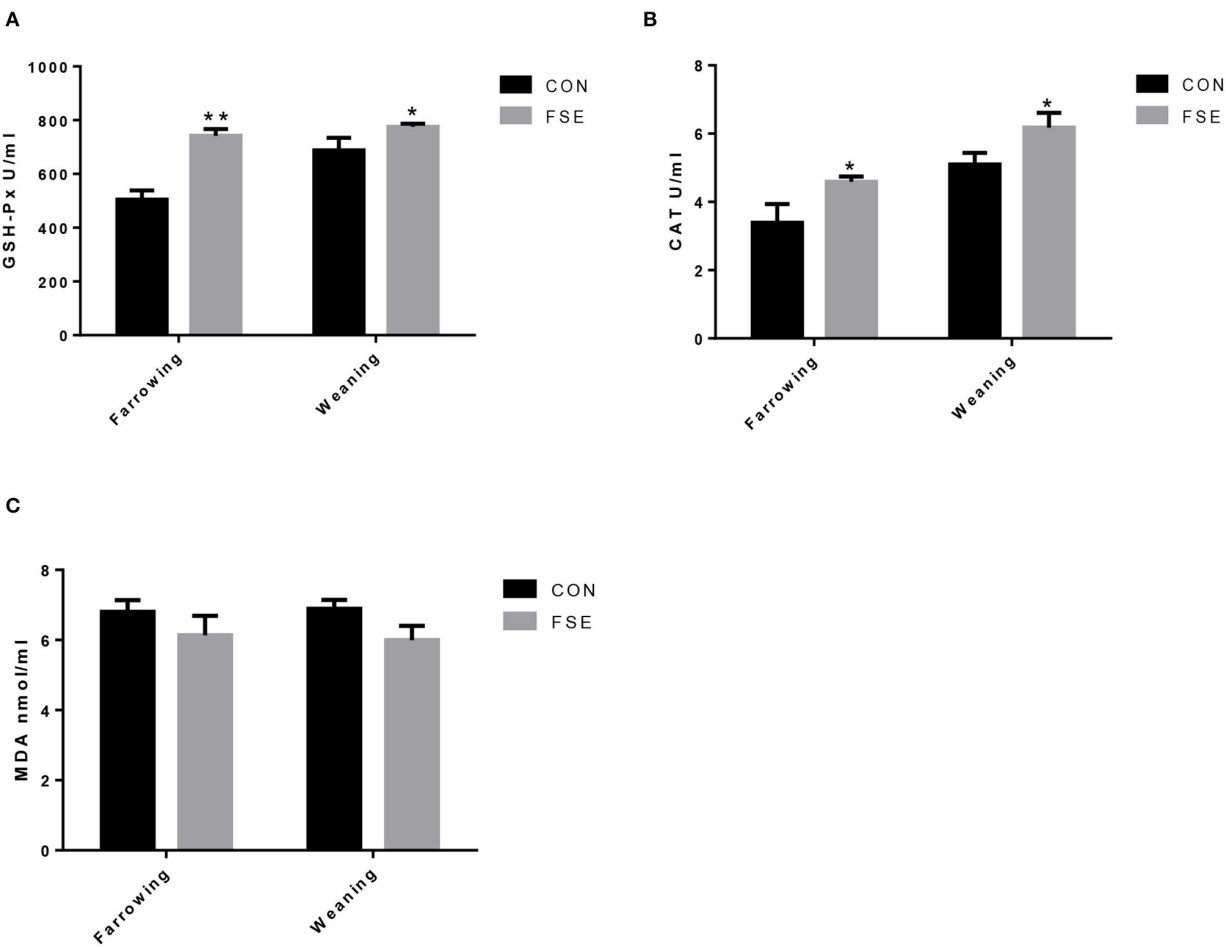
Effects of *Forsythia suspensa* extract on serum antioxidant status of lactating sows. CON, control treatment; FSE, *Forsythia suspensa* extract treatment; **(A)** GSH-Px, glutathione peroxidase; **(B)** CAT, catalase; **(C)** MDA, malondialdehyde. All values are expressed as means ± SEM (*n* = 12). *P < 0.05, **P < 0.01.

Compared with CON, sows fed FSE diet showed higher (*P* < 0.05) serum GSH-Px and CAT contents at farrowing. These sows also had higher (*P* < 0.05) serum GSH-Px and CAT contents at weaning (Figure 1).

**Figure 2:**
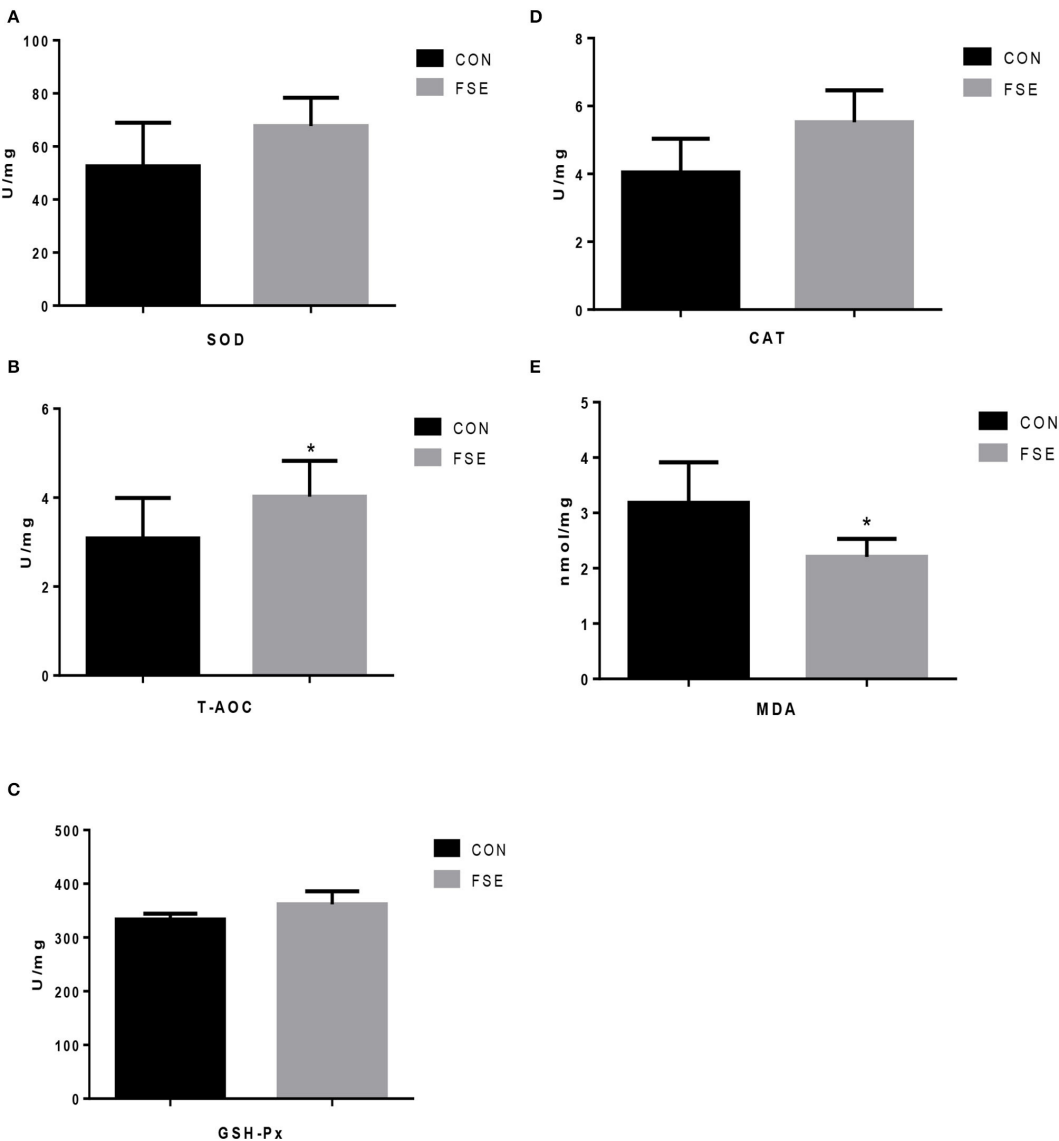
Effects of *Forsythia suspensa* extract on antioxidant status of placenta in lactating sows. CON, control treatment; FSE, *Forsythia suspensa* extract treatment; **(A)** SOD, superoxide dismutase; **(B)** T-AOC, total antioxidant capacity; **(C)** GSH-Px, glutathione peroxidase; **(D)** CAT, catalase; **(E)** MDA, malondialdehyde. All values are expressed as means ± SEM (*n* = 6). *P < 0.05.

As shown in Figure 2, compared with CON, sows supplemented with an FSE diet showed higher T-AOC levels and lower (*P* < 0.05) MDA levels in the placenta.

**Figure 3:**
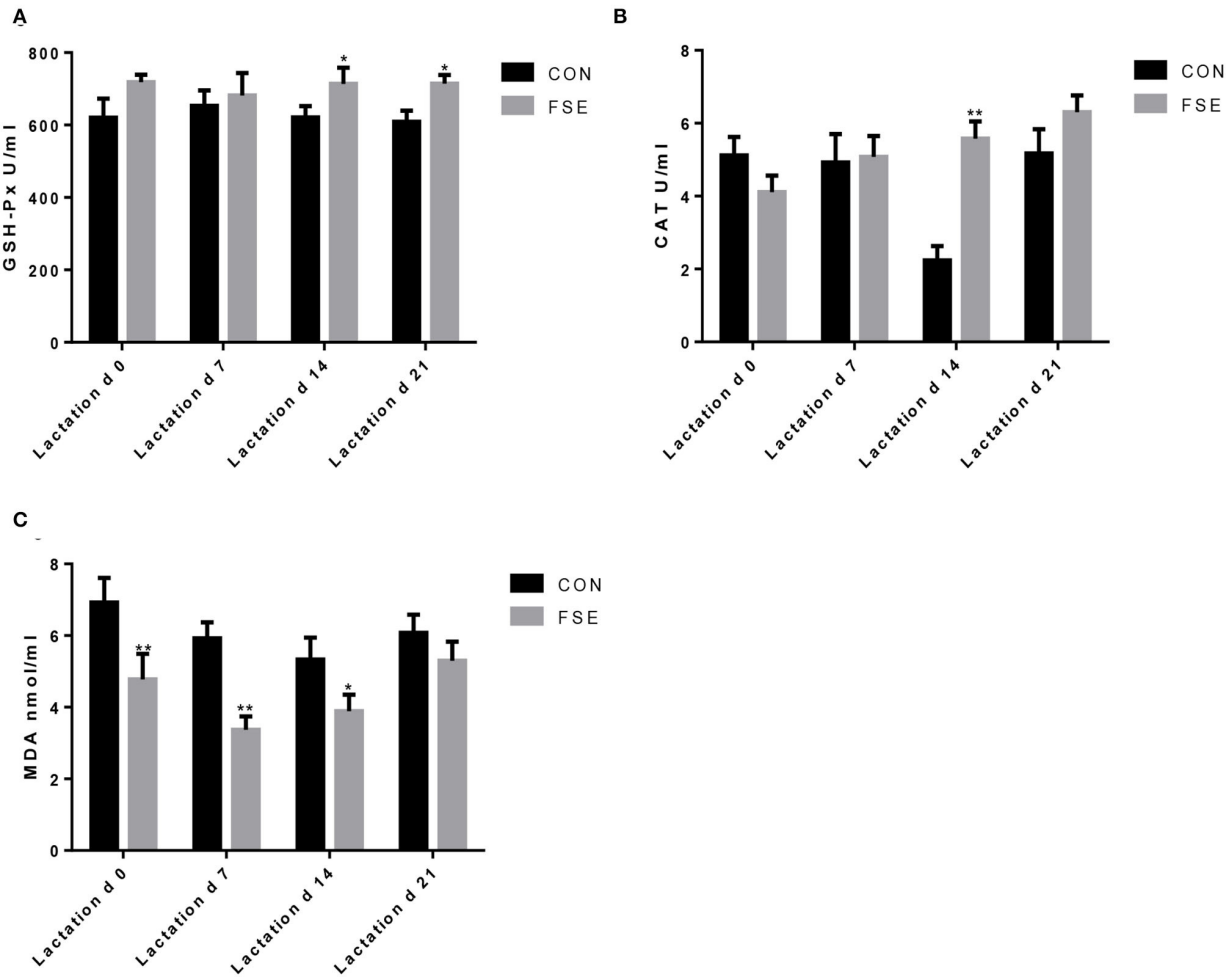
Effects of *Forsythia suspensa* extract on antioxidant capacity of suckling piglets. CON, control treatment; FSE, *Forsythia suspensa* extract treatment. **(A)** GSH-Px, glutathione peroxidase; **(B)** CAT, catalase; **(C)** MDA, malondialdehyde. All values are expressed as means ± SEM (*n* = 12). *P < 0.05, **P < 0.01.

As presented in Figure 3, the content of serum MDA was lower (*P* < 0.05) in suckling piglets of FSE-fed sows at farrowing compared with CON. On d 7 of lactation, the content of serum MDA was reduced (*P* < 0.05) in piglets of FSE-fed sows compared with CON. On d 14 of lactation, compared with CON, the contents of serum GSH-Px and CAT were increased (*P* < 0.05), while the content of serum MDA was reduced (*P* < 0.05) in piglets from FSE-fed sows. At weaning, compared with CON, the contents of serum GSH-Px were enhanced (*P* < 0.05) in piglets from FSE-fed sows.

### Interleukin Cytokines of Sows and Suckling Piglets

**Figure 4:**
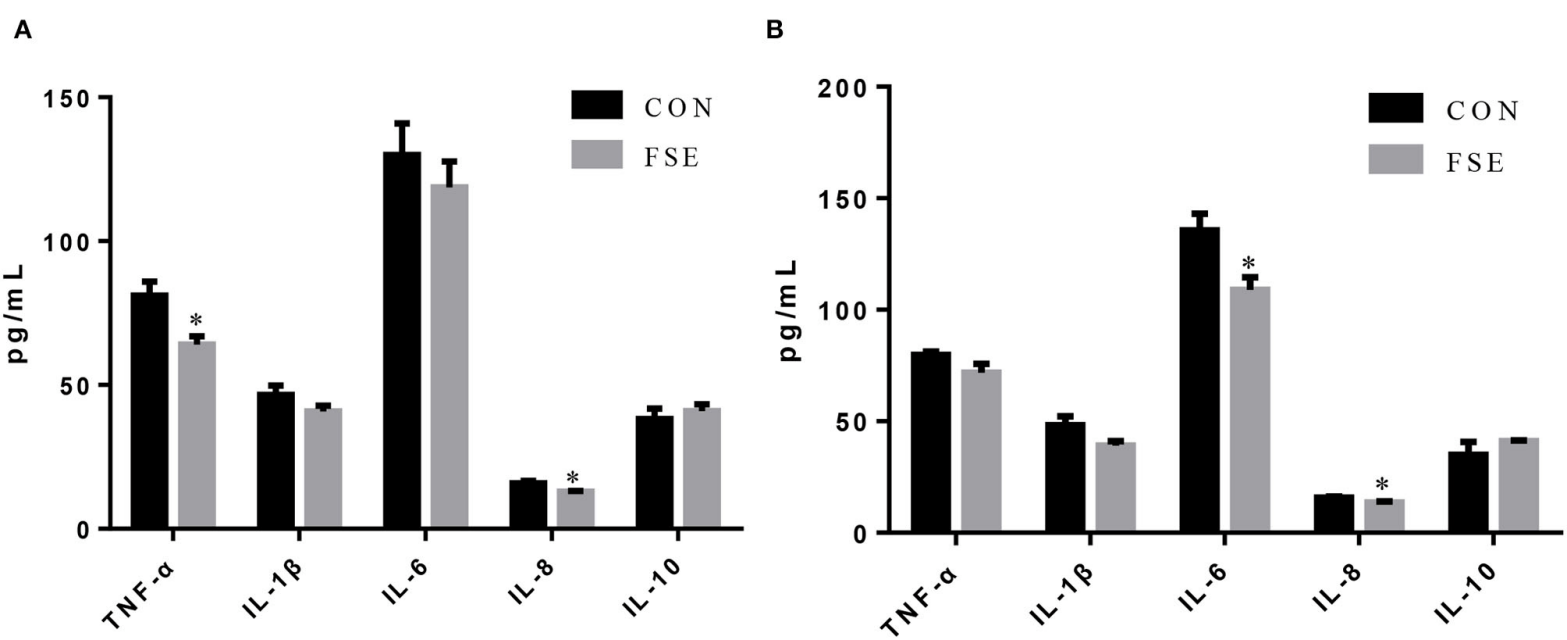
Effects of *Forsythia suspensa* extract on inflammatory cytokines in serum of lactating sows. CON, control treatment; FSE, *Forsythia suspensa* extract treatment. **(A)** Inflammatory cytokines in serum of sows at farrowing; **(B)** Inflammatory cytokines in serum of sows at weaning. All values are expressed as means ± SEM (*n* = 6). *P < 0.05.

As shown in Figure 4, compared with CON, sows fed FSE had lower (*P* < 0.05) TNF-α and IL-8 contents in serum at farrowing and decreased (*P* < 0.05) serum IL-8 and IL-6 contents at weaning.

**Figure 5:**
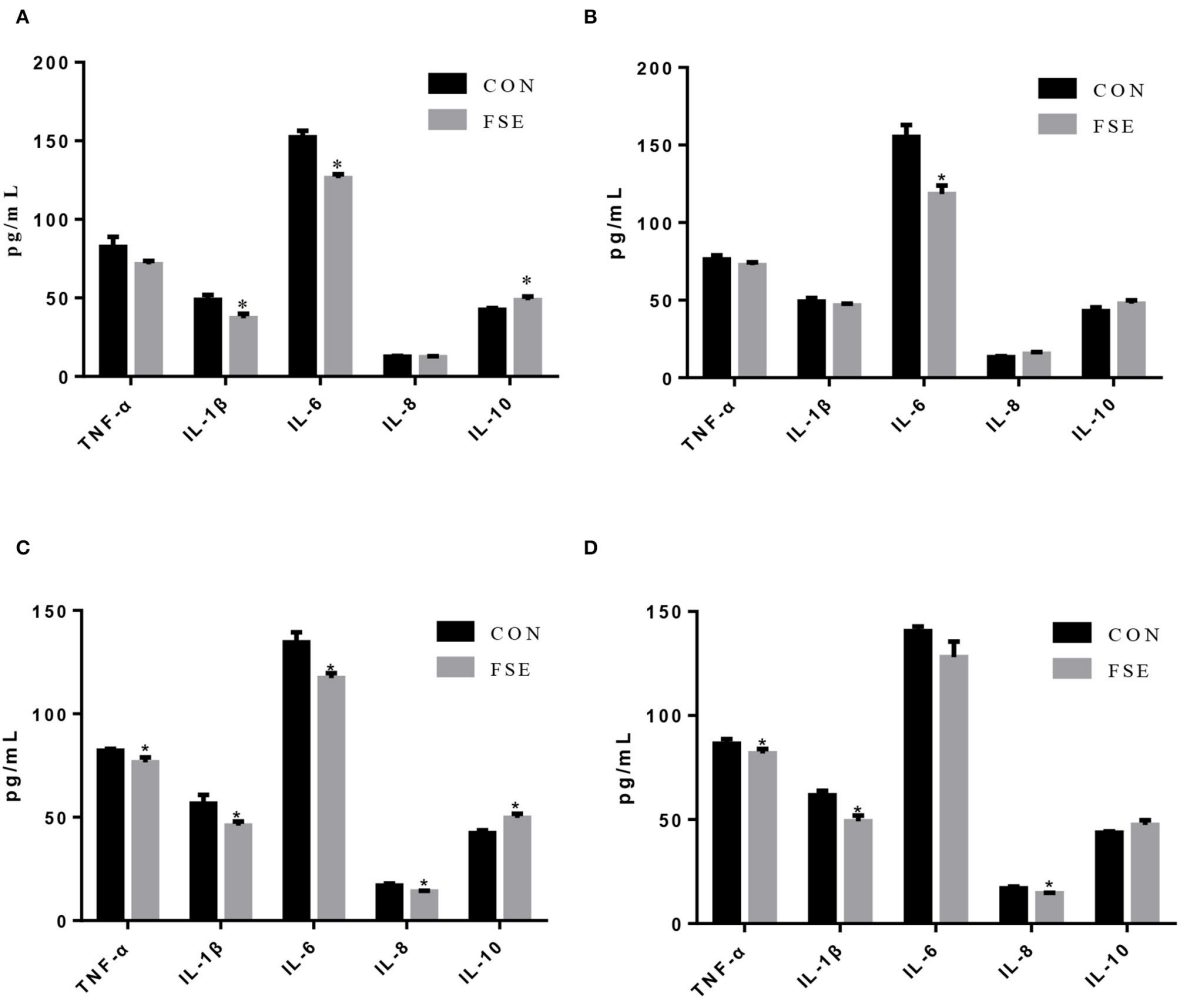
Effects of *Forsythia suspensa* extract on inflammatory cytokines in serum of suckling piglets. CON, control treatment; FSE, *Forsythia suspensa* extract treatment. **(A)** Inflammatory cytokines in serum of piglets at farrowing; **(B)** Inflammatory cytokines in serum of piglets on d 7 of lactation. **(C)** Inflammatory cytokines in serum of piglets on d 14 of lactation. **(D)** Inflammatory cytokines in serum of piglets at weaning. All values are expressed as means ± SEM (*n* = 6). *P < 0.05.

As shown in Figure 5, compared with CON, piglets from sows fed FSE had lower (*P* < 0.05) serum IL-1β and IL-6 contents and higher (*P* < 0.05) serum IL-10 content at farrowing. These piglets also had lower (*P* < 0.05) content of IL-6 in serum on d 7 of lactation compared with CON. On d 14 of lactation, piglets from sows supplemented with FSE had lower (*P* < 0.05) TNF-α, IL-1β, IL-6, and IL-8 contents and higher (*P* < 0.05) IL-10 content in serum compared with CON. On d 21 of lactation, compared with CON, piglets from sows fed FSE had lower (*P* < 0.05) serum TNF-α, IL-1β, and IL-8 contents.

### The mRNA Expression of Antioxidant Enzymes in Suckling Piglets

**Figure 6:**
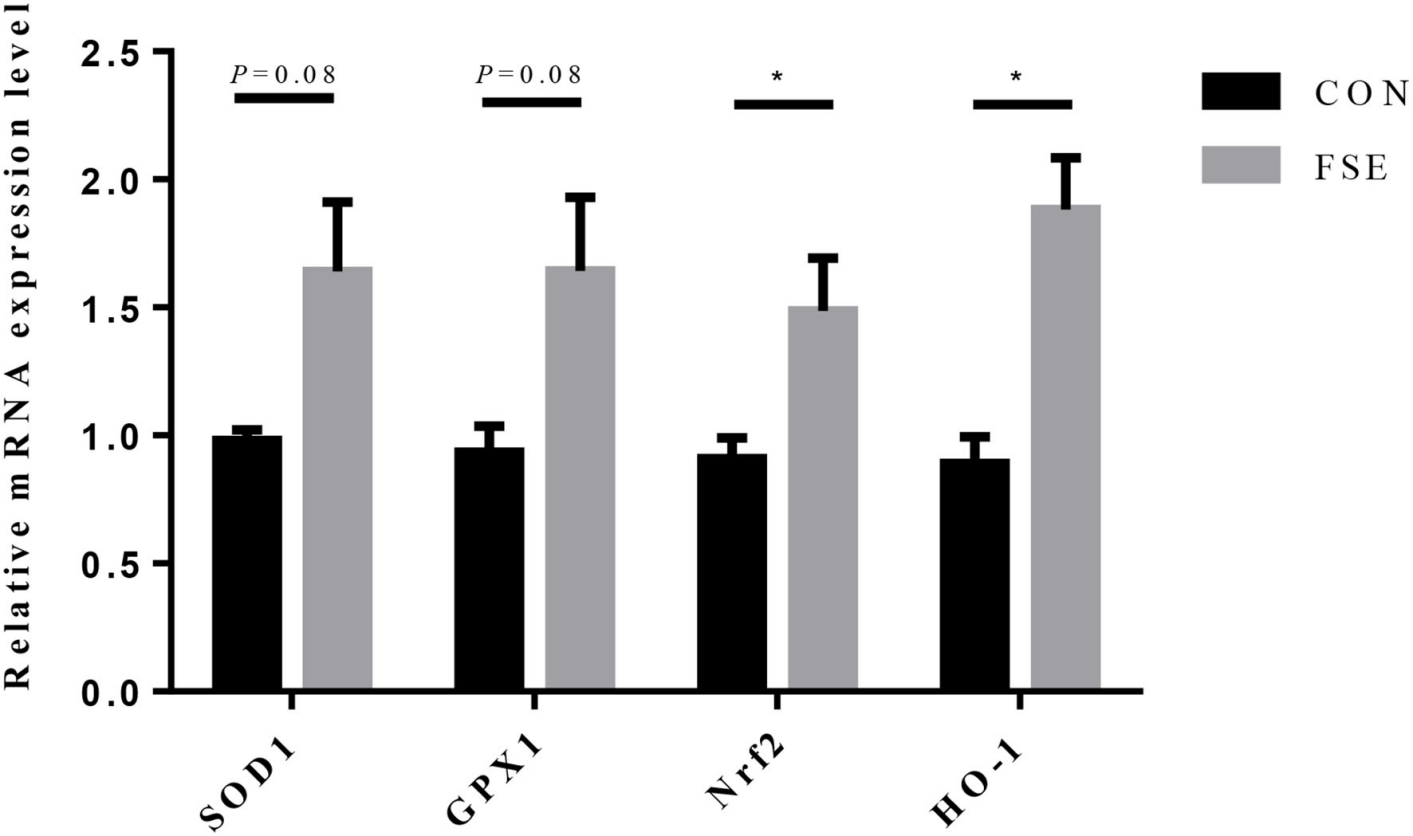
Effects of maternal dietary *Forsythia suspensa* extract supplementation on relative mRNA expression of antioxidant genes in liver of suckling piglets. CON group, basal diet; FSE group, basal diet + 100 mg/kg *Forsythia suspensa* extract. All values are expressed as means ± SEM. *P < 0.05. *n* = 6.

For piglets of FSE-fed sows, the gene expression of Nrf2 and HO-1 were increased (*P* < 0.05), while the gene expression of SOD1 and GPx1 tended to be increased (*P* = 0.08) in the liver compared with CON (Figure 6).

### Intestinal Morphology and Gut Barrier Function in Suckling Piglets

At weaning, piglets from FSE-fed sows had enhanced (*P* ≤ 0.05) villus height to crypt depth ratio in duodenum and jejunum, higher (*P* < 0.05) villus height in the jejunum, and tended to have increased villus height in the duodenum (*P* = 0.10) and ileum (*P* = 0.08) compared with CON (Table 3).

**Table 3:** Effects of *Forsythia suspensa* extract on intestinal morphology of suckling piglets.

	**Control**	***Forsythia suspensa* extract**	**SEM**	***P*-Value**
**Duodenum**				
Villus height, μm	444	602	59.26	0.10
Crypt depth, μm	277	305	22.22	0.42
Villus height/Crypt depth	1.59	1.98	0.10	0.04
**Jejunum**				
Villus height, μm	313	490	38.86	0.02
Crypt depth, μm	171	223	20.43	0.13
Villus height/Crypt depth	1.83	2.21	0.11	0.05
**Ileum**				
Villus height, μm	306	364	18.33	0.08
Crypt depth, μm	171	176	5.29	0.50
Villus height/Crypt depth	1.77	2.11	0.12	0.12

*SEM, standard error of means. n = 6*.

**Figure 7:**
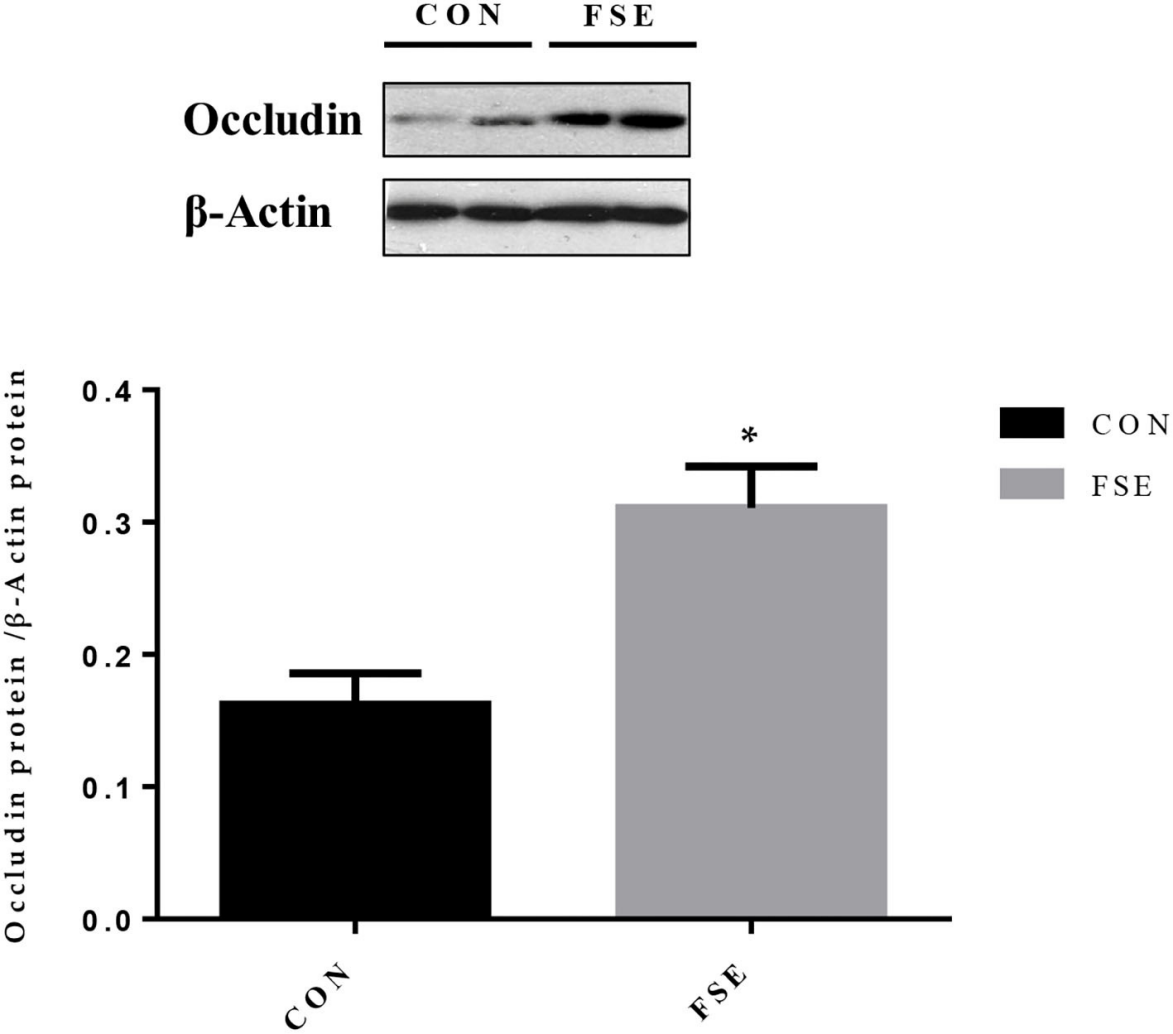
Effects of maternal dietary *Forsythia suspensa* extract supplementation on protein expression of occludin proteins in jejunum mucosa of suckling piglets. CON group, basal diet; FSE group, basal diet + 100 mg/kg *Forsythia suspensa* extract. All values are expressed as means ± SEM. *P < 0.05. *n* = 6.

Moreover, piglets from sows supplemented with FSE had higher (*P* < 0.05) protein expression of occludin in jejunal mucosa at weaning (Figure 7).

### Intestinal VFA and Microbiota Composition in Lactating Sows or Suckling Piglets

Compared with CON, the contents of formic acid and isobutyric acid were increased (*P* ≤ 0.05) in the cecum of suckling piglets from FSE-fed sows. The content of propionic acid was increased (*P* < 0.05), while the contents of isobutyric acid and total VFA (TVFA) tend to be improved (*P* = 0.09) in the colon of piglets from FSE-fed sows compared with CON (Table 4).

**Table 4:** Effects of maternal dietary *Forsythia suspensa* extract supplementation on the contents of total volatile fatty acid in digesta of suckling piglets (mg/kg).

**Items**	**CON**	**FSE**	**SEM**	***P*-Value**
**Cecum**				
Formic acid	0.15	0.22	0.39	0.05
Acetic acid	7.28	6.95	0.92	0.07
Propionic acid	4.74	6.87	0.44	0.70
Butyric acid	3.20	2.11	0.44	0.88
Isobutyric acid	0.04	0.08	0.30	0.02
Valeric acid	0.20	0.41	0.51	0.19
Isovaleric acid	0.02	0.02	0.01	1.00
Total volatile fatty acid	15.63	16.66	4.73	0.94
**Colon**				
Formic acid	0.13	0.17	0.02	0.21
Acetic acid	4.23	6.98	1.19	0.20
Propionic acid	2.53	4.23	0.20	<0.01
Butyric acid	0.92	1.52	0.23	0.17
Isobutyric acid	0.05	0.07	0.01	0.09
Valeric acid	0.02	0.04	0.01	0.34
Isovaleric acid	0.08	0.13	0.03	0.30
Total volatile fatty acid	7.96	13.15	1.50	0.09

*SEM, standard error of means. n = 6*.

*CON, Control; FSE, Forsythia suspensa extract*.

**Figure 8:**
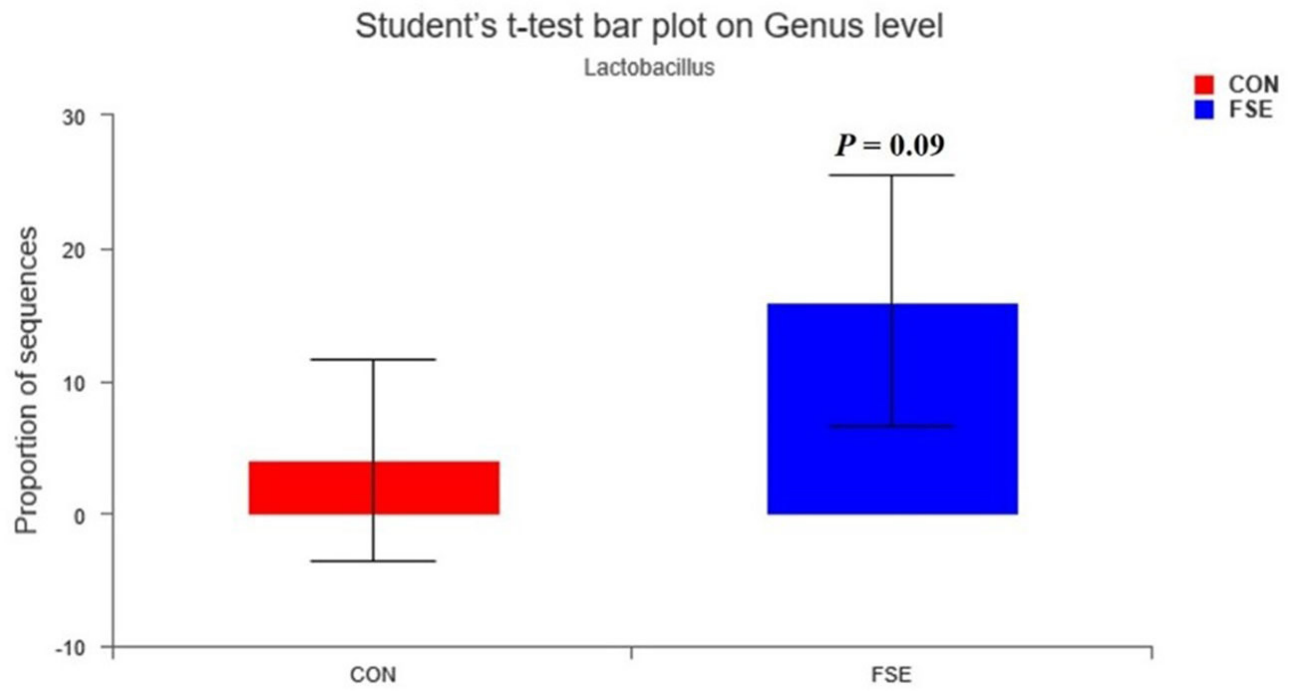
Effects of *Forsythia suspensa* extract fed to sows on *Lactobacillus* community in feces of sows. The different colors represented the different treatments. The results presented as mean values of the bacteria, *n* = 4. CON, control; FSE, *Forsythia suspensa* extract.

**Figure 9:**
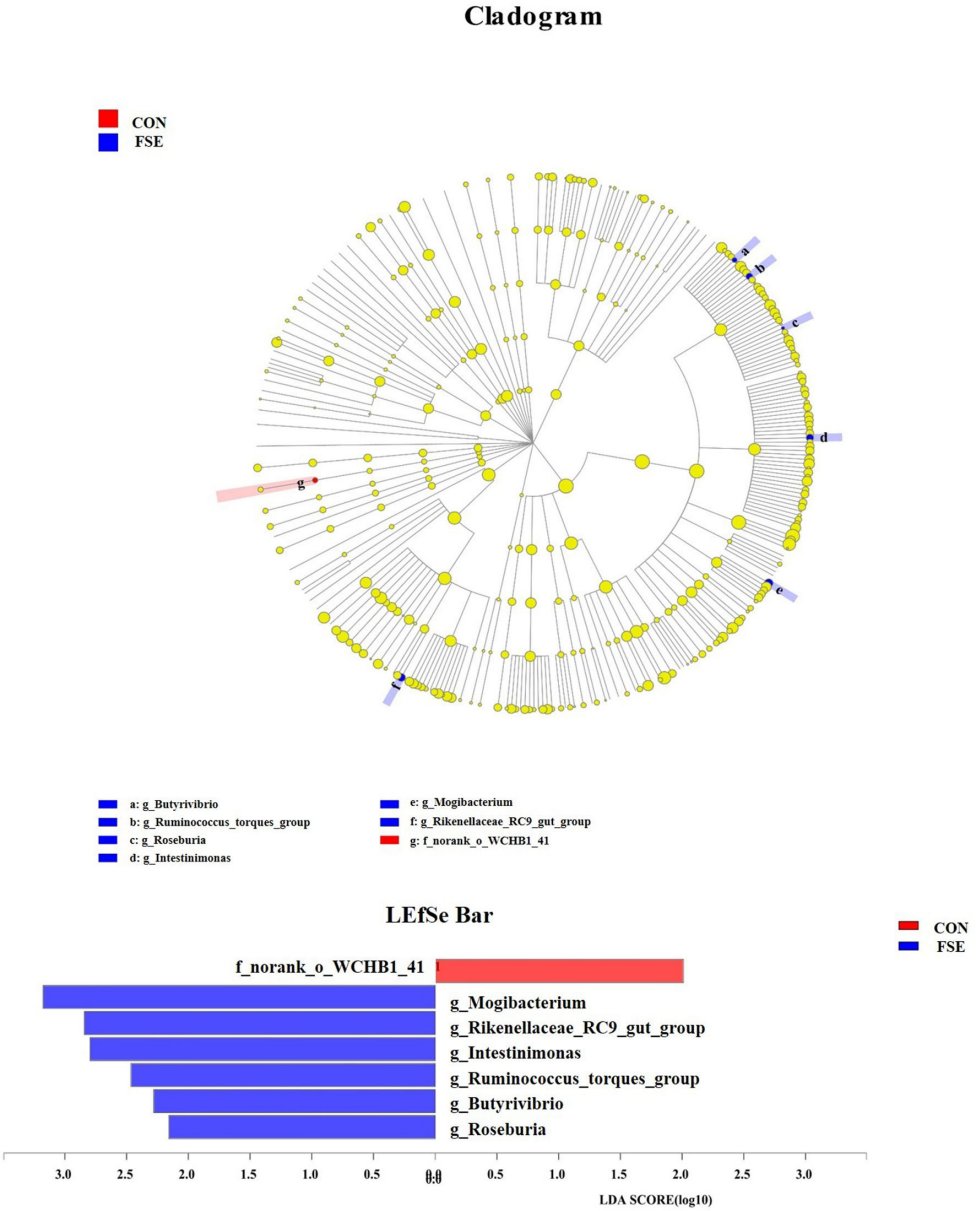
The analysis for different bacteria communities from phylum to genus level in the cladogram of LEfSe among treatments. Relative abundance of bacteria communities from phylum to genus level in feces of sows. CON, control; FSE, *Forsythia suspensa* extract. *n* = 4.

As shown in Table 5, there was no significant difference in α-diversity in sows and piglets between the two treatments. As shown in Figures 8, 9, in the feces of sows, sows fed FSE tended to have increased (*P* = 0.09) the relative abundance of *Lactobacillus* and increased (*P* < 0.05) the relative abundance of *Mogibacterium, Rikenellaceae_RC9_gut_group, Intestinimonas, Ruminococcus_torques_group, Butyrivibrio*, and *Roseburia* at the genus level.

**Table 5:** Effects of maternal dietary *Forsythia suspensa* extract supplementation on α-diversity in cecum and colon microbiota of fecal microbiota of sows.

**Items**	**CON**	**FSE**	**SEM**	***P-*Value**
Sobs	324.75	377.25	100.72	0.49
Shannon	2.34	3.12	0.56	0.12
Simpson	0.30	0.14	0.11	0.13
Ace	404.71	437.67	99.82	0.66
Chao	406.61	446.34	110.55	0.63

*SEM, standard error of means. n = 4*.

*CON, Control; FSE, Forsythia suspensa extract*.

## Discussion

During lactation, sows and piglets might face with oxidative stress, which could lead to poor antioxidant status, immunity, and reproductive performance in sows, and inhibit and impair the growth and health status in suckling piglets (3). Moreover, high-productive sows might also face severe backfat and BW losses during lactation, which might reduce the sow's longevity and reproductive efficiency. Therefore, it is important to find a solution to reduce their body tissue losses and increase the feed intake of lactating sows. The FSE is a kind of traditional Chinese medicine herb, which has been showing potentially effective in increasing ADFI and ADG in weaned piglets and broilers (14, 22), indicating that 100 mg/kg FSE could lead to better appetite in animals. Moreover, under heat-induced oxidative stress conditions, FSE could also improve ADFI and ADG of animals (9), which indicated the beneficial effects of FSE on alleviating stresses. In the current study, we found sows fed FSE diet showed an increase of ADFI by 2% and lower the BW loss from farrowing to weaning, which demonstrated the beneficial effect of FSE on alleviating the oxidative stress-induced BW loss in sows. The bioactivity-guided fractionation of the methanol extract of the fruits of *Forsythia suspensa* Vahl contained forsythialan A, B, and phillyrin (37). The FSE in this study mainly contained forsythiaside A, forythialan A, phillygenin, and phillyrin, while the phenolic hydroxyl in these components of FSE could act as hydrogen donor and play an effective role in scavenging a variety of ROS (38). Moreover, this finding might also be due to the beneficial effects of these polyphenols in FSE on reacting with metal ions, such as iron ions and copper ions, which could promote the generation of ROS, thus protecting the animals from oxidative stress (39). In this study, FSE could also decrease the WEI in lactating sows, which might be due to the beneficial effect of FSE on maintaining the BW of sows and partly help improve the PSY of sows.

In the present study, we also found the improvement of ADG on d 7–21 of lactation in suckling piglets of FSE-fed sows, which proved that maternal FSE supplementation could be used to improve performance in their offspring. This finding might be due to the effect of polyphenol in forythialan A, forsythiaside A, phillygenin, and phillyrin on improving immunity and antioxidant capacity in pigs. Moreover, similar to the current finding, previous studies in our lab also demonstrated that FSE could lead to better ADG of weaned piglets or broilers, especially in the later phases, which was mainly due to its accumulation effect and beneficial effects on improving nutrient digestibility, immunoglobulin, and intestinal health (14, 23). The appearance of oxidative stress in the perinatal period of animals made the energy used for synthesizing milk or other production turn to the synthesis of antioxidant substances, which reduced the production efficiency and the quality of milk due to the accumulation of oxidative substances (40). Since the colostrum and milk were the only resources of energy, protein, and other nutrients for suckling piglets, the quality of colostrum and milk from sows played an important role in the growth and health status of suckling piglets from birth to weaning. A previous study showed that maternal FSE supplementation in sows could increase the milk fat content on d 7 of lactation, indicating that FSE might increase the growth performance of piglets by improving the milk quality (41). Long et al. (24) also reported that FSE could increase the apparent total tract digestibility of gross energy, crude protein, and ether extract in gestating sows, which might provide piglets with more lactose, protein, fat, and other nutrients, and contribute to the enhanced ADG in piglets in the current study.

When oxidants and antioxidants were in an unbalanced state in body tissue, a large number of ROS could be produced. The Fe^2+^ and Cu^2+^ in organisms could make cells react in the process of aerobic metabolism to produce hydroxyl radicals. These excessive ROS and free radicals could lead to lipid peroxidation, DNA damage, and even cell death (42). The protective effect of forsythiaside A, forythialan A, and phillygenin in FSE (100 mg/kg BW) against oxidative stress was better than vitamin C, indicating that FSE could effectively reduce free radical *in vitro* (11). Long et al. (24) reported that FSE could improve the antioxidant capacity in serum of gestating sows and newborn piglets at farrowing, indicating that FSE might effectively reduce oxidative stress *in vivo*. To figure out the effect and possible maternal antioxidant transmission pathway of FSE on modulating antioxidant capacity of lactating sows and their offspring, we measured the parameter for antioxidant enzymes system (including GSH-Px and CAT) and lipid oxidation marker (MDA) in serum of sows, placenta, and serum of piglets from farrowing to weaning. We found sows fed FSE had enhanced serum CAT and GSH-Px contents at farrowing and weaning, as well as reduced MDA content in the placenta compared with CON. While the suckling piglets of FSE-fed sows had increased serum GSH-Px contents at weaning, as well as lower serum MDA content on d 0, 7, and 14 of lactation. One of the possible reasons for the current finding might be the forsythiaside in FSE could activate antioxidant enzymes system (such as CAT and GSH-Px) *via* upregulating the Nrf2 level in the nucleus, and therefore prevent cell apoptosis and reducing ROS and MDA generation (43). Furthermore, this finding might also be due to the beneficial effect of polyphenols in FSE on regulating antioxidant gene expression in the placenta by the Keap1-Nrf2 pathway and Sirt1, leading to increased GSH-Px and CAT levels in serum of sows and piglets (44).

Li et al. (45) reported that traditional Chinese medicine could increase the SOD and GSH contents and reduce MDA level and cell apoptosis *via* upregulating the Nrf2/HO-1 signaling pathway. To reveal the possible mechanism of FSE in modulating the antioxidant capacity of lactating sows and their offspring, we measured the mRNA expression of Nrf2, HO-1, SOD1, and GPx1 in the liver of suckling piglets at weaning. The upregulation of Nrf2 and HO-1 genes could alleviate oxidative stress, while the HO-1 was also proved effective in increasing anti-inflammatory function (46, 47). The SOD1 (CuZn-SOD) could regulate functions in growth, metabolism, and oxidative stress responses (48). The GPx1 could reduce the alkyl hyperoxides and hydrogen peroxide (49), while the overexpression of GPx1 was beneficial to protect mice against oxidative stress (50). Currently, we found the mRNA expression of Nrf2 and HO-1 was enhanced, while the mRNA expression of SOD1 and GPx1 tended to be increased in the liver in piglets of FSE-fed sows, which supported the improved levels of antioxidant enzyme activities in the serum of piglets. This finding revealed that the Nrf2/HO-1 signaling pathway might play a protective role in FSE in alleviating oxidative stress in sows and their offspring because of its antioxidant and anti-inflammatory functions (51). Thus, based on previous and current findings, we suspected that FSE might activate the Nrf2/HO-1 signaling pathway, increase the mRNA expression of SOD1 and GPx1, and thus enhance the levels of antioxidant enzymes (including SOD and GSH-Px) in serum of sows and their offspring. We also speculated the possible maternal antioxidant transmission pathway of FSE on modulating antioxidant capacity might be from the serum or milk of sows to the liver and serum of suckling piglets.

Oxidative stress and inflammation played critical roles in the health status of livestock. Lu et al. (11) reported that 25, 50, and 100 mg/kg FSE could effectively substitute 100 mg/kg vitamin C in lowering plasma TNF-α, IL-1β, and IL-6 levels *in vivo*. Previous studies also showed that FSE could alleviate the negative effects of corticosterone (15) or anaphylactic symptoms induced by β-conglycinin (10), which indicated that FSE might potentially play an important role in inflammatory responses. The current study showed that sows had lower serum IL-8 and TNF-α contents at farrowing and decreased serum IL-8 and IL-6 contents at weaning, while their offspring had lower serum IL-6 content on d 0, 7, and 14 of lactation and decreased serum IL-1β level on d 14 and 21 of lactation. The reason for this finding might be that the forsythiaside A in FSE could reduce inflammatory responses in cells *via* inhibiting NF-κ B and Nrf2/HO-1 signaling pathway activation (52). Besides, this finding might also be due to that the forsythiaside in FSE could also decrease the contents of local and systemic inflammatory mediators (53) and inhibit the activation of NF-?B, which might help detoxifying and heat-clearing in lactating sows and their offspring (54). Another possible reason for the modulation of anti-inflammatory factors was that the FSE could inhibit the Nrf2-mediated anti-inflammatory response (13). The current study also found that piglets from sows fed FSE had higher serum IL-10 (one of the anti-inflammatory cytokines) content at farrowing and on d 14 of lactation, which might be due to that the forsythiaside in FSE could improve the anti-inflammatory function *via* the activation of NF-κ B signaling pathway (55). Therefore, we suspected that FSE might improve the anti-inflammatory function in sows and suckling piglets *via* regulating the NF-κB signaling pathway, reducing pro-inflammatory cytokines (IL-1β and IL-6), or increasing anti-inflammatory cytokines (IL-10).

Stress might also lead to the impairment of gut morphology and barrier function (56). For the suckling piglets, the development of gut morphology was important for their utilization of nutrients (including protein, lactose, lipid, and minerals) from milk. Han et al. (22) reported the positive effects of FSE on improving villus height and villus height to crypt depth ratios in both duodenum and ileum. Long et al. (23) also pointed out that the FSE could enhance the villus height to crypt depth ratio in the ileum of piglets. The current study showed that maternal dietary FSE supplementation could modulate gut morphology by increasing the villus height and the villus height to crypt depth ratio in the duodenum and jejunum. This finding was mainly due to that the FSE could repair epidermis damage caused by antigens and pathogens in feed (22), as well as modulate intestinal permeability and alleviate intestinal injury in piglets (13). Epithelial barrier function was important for nutrient absorption, while the occludin protein was one of the main conjunction proteins (57). Our previous study also showed that maternal FSE supplementation could increase the occludin protein expression in their piglets after weaning for 28 days (41), while in the current study, the protein expression of occludin was increased in FSE-fed suckling piglets, indicating the absorption of nutrients in milk might be improved, which was beneficial for the intestinal development and health status of suckling piglets. The improved barrier function by Chinese medicine (Tong-fu-li-fei, containing FSE) might be due to the increased protein expression of zona occludens 1/occludin/claudin-1 (58).

The VFA played an important role in solving some gut diseases. Feng et al. (59) reported that the herb medicines could regulate the composition of TVFA *via* modulating the gut microbial community. In the current study, we found the contents of formic acid and isobutyric acid were increased in cecum piglets from FSE-fed sows. The content of propionic acid was increased, while the contents of isobutyric acid and TVFA tended to be increased in the colon of piglets from FSE-fed sows, indicating that gut VFA could be modulated by FSE. The propionic acid could be used as the intermediate of succinyl COA and methylmalonyl COA (60). The VFA produced by bacterial fermentation was an important component of colonic contents, which could be rapidly absorbed by colonic epithelial cells and participate in metabolism and supply energy (61), while the organic acids in the large intestine could also help enhance the immunity and regulate the micro-ecological environment in the digestive tract of piglets (31, 62). Therefore, the trend for the increased TVFA content in the colon was beneficial for the immunity and gut health of suckling piglets in the current study.

The intestinal microbiota was crucial to the health status *via* providing metabolic, immunologic, and protective functions (63). Many methods could be used to modulate the intestinal microbial composition of animals. Dietary *B. subtilis* supplementation could improve microbial community and growth performance of animals *via* increasing abundance of *Blautia, Faecalibacterium, Flavonifractor, Hydrogenoanaerobacterium*, and *Romboutsia* (64). Besides, some enzymes, such as IONzymes, had antibacterial effects and could reduce *S. enteritidis* infections in animals (65). Moreover, many plant extracts, such as isoflavones exert, also had protective effects on the intestinal health of animals (66). In the present study, sows fed FSE tended to have a higher relative abundance of *Lactobacillu*s at genus level in feces at weaning, the beneficial bacteria might pass from sows to their offspring, which was possibly a pathway of maternal FSE on modulating gut barrier function and health status in suckling piglets (67). This finding helped explain the improved immunity, anti-inflammatory functions, and ADG in piglets, since *Lactobacillus*, serving as a probiotic, could effectively increase the expression of IL-10 and TGF-β and decrease the expression of some pro-inflammatory cytokines (such as TNF-α and IL-6) in the colon (68), and thus decreased diarrhea and improved performance in suckling piglets (69). This finding might be due to that the forsythiaside in FSE could enhance the levels of *Bifidobacteria, Lactobacillus*, and other beneficial microbial community (10). Long and Piao (41) also showed that maternal FSE supplementation could also increase the relative abundance of *Lactobacillus* in their piglets after weaning for 28 days, indicating maternal FSE supplementation might also have a beneficial effect on suckling piglets. Moreover, previous studies also reported that FSE could modulate the microbial community in the large intestine of broilers (22) and piglets (23), which might also be due to the beneficial effect of essential oil from FSE (70).

The current study also found that FSE increased the relative abundance of *Ruminococcus_torques_group, Butyrivibrio*, and *Roseburia* at the genus level in the sows. *Roseburia* spp. could effectively produce VFA (e.g., butyrate or butyric acid) and could be beneficial probiotics for gut health (71). The increased relative abundance of *Ruminococcus_torques_group* indicated that some fermentative microbiota were modulated by FSE and seemed to promote better maternal intestinal microbial sources for piglets. *Butyrivibrio* could ferment glucose to produce butyrate, which was beneficial for supplying energy for the gut cell and the growth of beneficial bacteria (31, 72). The reason for the current findings might be that Chinese medicine could help decrease colonic or caecal pH levels, enhance the abundance of the beneficial microbial community, and colonic inflammation response *via* downregulating the TLR4/MyD88/NF-κB signaling pathway and reducing mRNA expression of some pro-inflammatory cytokines (73). The current findings indicated that the maternal FSE could influence the composition of the intestinal microbiota in sows while its mechanism still needed to be further investigated.

## Conclusion

In conclusion, dietary FSE supplementation improved reproductive performance, antioxidant status, inflammatory responses, and gut microbiota composition in lactating sows. Besides, maternal dietary FSE supplementation could also improve the antioxidant capacity, anti-inflammatory function, intestinal development (*via* improving intestinal morphology and barrier function), and health status (*via* improving gut volatile fatty acid) in suckling piglets. The result of this study suggested that maternal dietary FSE supplementation could be a beneficial way to solve the oxidative stress problem in sow production during late gestation and lactation periods.

## Data Availability Statement

The data presented in the study are deposited in the NCBI repository, accession number is PRJNA848636.

## Ethics Statement

The animal study was reviewed and approved by Institutional Animal Care and Use Committee of China Agricultural University (No. AW52301202-1-1, Beijing, China).

## Author Contributions

SLo, QW, TH, and XP: conceptualization. SLo and JM: methodology. SLo and JW: software. SLo: validation, formal analysis, investigation, resources, data curation, and writing—original draft preparation. HW, LL, and XP: writing—review and editing. XP: supervision, project administration, and funding acquisition. All authors contributed to the article and approved the submitted version.

## Funding

This research was funded by Beijing Municipal Natural Science Foundation (6202019) and the National Key Research and Development Program of China (2021YFD1300201).

## Conflict of Interest

LL was employed by Tianjin Zhongsheng Feed Co., Ltd. The remaining authors declare that the research was conducted in the absence of any commercial or financial relationships that could be construed as a potential conflict of interest.

## Publisher's Note

All claims expressed in this article are solely those of the authors and do not necessarily represent those of their affiliated organizations, or those of the publisher, the editors and the reviewers. Any product that may be evaluated in this article, or claim that may be made by its manufacturer, is not guaranteed or endorsed by the publisher.

## References

[1] Agarwal A Gupta S Sharma RK. Role of oxidative stress in female reproduction. Reprod Biol Endocrin. (2005) 3:28. 10.1186/1477-7827-3-28 16018814 PMC1215514

[2] Yin J Ren W Liu G Duan J Yang G Wu L. Birth oxidative stress and the development of an antioxidant system in newborn piglets. Free Radic Res. (2013) 47:1027–35. 10.3109/10715762.2013.848277 24074241

[3] Hu CH Xiao K Luan ZS Song J. Early weaning increases intestinal permeability, alters expression of cytokine and tight junction proteins, and activates mitogen-activated protein kinases in pigs. J Anim Sci. (2013) 91:1094–101. 10.2527/jas.2012-5796 23230104

[4] Theil PK Lauridsen C Quesnel H. Neonatal piglet survival: impact of sow nutrition around parturition on fetal glycogen deposition and production and composition of colostrum and transient milk. Animal. (2014) 8:1021–30. 10.1017/S1751731114000950 24762853

[5] Leonard SG Sweeney T Bahar B Lynch BP O'Doherty JV. Effect of dietary seaweed extracts and fish oil supplementation in sows on performance, intestinal microflora, intestinal morphology, volatile fatty acid concentrations and immune status of weaned pigs. Br J Nutr. (2011) 105:549–60. 10.1017/S0007114510003739 20875191

[6] Xie C Long C Wu X Yang H Fan Z Xiao D. Effect of maternal supplementation with chitosan oligosaccharide on the antioxidant capacity of suckling piglets. J Anim Sci. (2016) 94:453–6. 10.2527/jas.2015-9611

[7] Kim SW Weaver AC Shen YB Zhao Y. Improving efficiency of sow productivity: nutrition and health. J Anim Sci Biotechnol. (2013) 4:2–9. 10.1186/2049-1891-4-26 23885840 PMC3733949

[8] Thakur GS Bag M Sanodiya BS Bhadauriya P Debnath M Prasad GBKS. *Momordica balsamina*: a medicinal and neutraceutical plant for health care management. Curr Pharm Biotechnol. (2009) 10:667–82. 10.2174/138920109789542066 19751180

[9] Wang L Piao XL Kim SW Piao XS Shen YB Lee HS. Effects of *Forsythia suspensa* extract on growth performance, nutrient digestibility, and antioxidant activities in broiler chickens under high ambient temperature. Poultry Sci. (2008) 87:1287–94. 10.3382/ps.2008-00023 18577607

[10] Hao Y Li DF Piao XL Piao XS. Forsythia suspensa extract alleviates hypersensitivity induced by soybean β-conglycinin in weaned piglets. J Ethnopharmacol. (2010) 128:412–8. 10.1016/j.jep.2010.01.035 20083183

[11] Lu T Piao XL Zhang Q Wang D Piao XS Kim SW. Protective effects of *Forsythia suspensa* extract against oxidative stress induced by diquat in rats. Food Chem Toxicol. (2010) 48:764–70. 10.1016/j.fct.2009.12.018 20036301

[12] Long SF He TF Wu D Yang M Piao XS. Forsythia suspensa extract enhances performance via the improvement of nutrient digestibility, antioxidant status, anti-inflammatory function, and gut morphology in broilers. Poultry Sci. (2020) 99:4217–26. 10.1016/j.psj.2020.05.011 32867965 PMC7598019

[13] Zhao PF Piao XS Zeng ZK Li P Xu X Wang HL. Effect of *Forsythia suspensa* extract and chito-oligosaccharide alone or in combination on performance, intestinal barrier function, antioxidant capacity and immune characteristics of weaned piglets. Anim Sci J. (2017) 88:854–62. 10.1111/asj.12656 27758020

[14] Zhang HY Piao XS Zhang Q Li P Yi JQ Liu JD. The effects of *Forsythia suspensa* extract and berberine on growth performance, immunity, antioxidant activities, and intestinal microbiota in broilers under high stocking density. Poultry Sci. (2013) 92:1981–8. 10.3382/ps.2013-03081 23873544

[15] Zeng ZK Li QY Piao XS Liu JD Zhao PF Xu X. Forsythia suspensa extract attenuates corticosterone-induced growth inhibition, oxidative injury, and immune depression in broilers. Poultry Sci. (2014) 93:1774–81. 10.3382/ps.2013-03772 24864291

[16] Zhao PF Piao XS Pan L Zeng ZK Li QY Xu X. Forsythia suspensa extract attenuates lipopolysaccharide-induced inflammatory liver injury in rats via promoting antioxidant defense mechanisms. Anim Sci J. (2017) 88:873–81. 10.1111/asj.12717 27753186

[17] Pan L Ma XK Zhao PF Shang QH Long SF Wu Y. Forsythia suspensa extract attenuates breast muscle oxidative injury induced by transport stress in broilers. Poultry Sci. (2018) 97:1554–63. 10.3382/ps/pey012 29528452

[18] Leonard SG Sweeney T Bahar B Lynch BP O'doherty JV. Effect of maternal fish oil and seaweed extract supplementation on colostrum and milk composition, humoral immune response, and performance of suckled piglets. J Anim Sci. (2010) 88:2988–97. 10.2527/jas.2009-2764 20453086

[19] Leonard SG Sweeney T Bahar B O'Doherty JV. Effect of maternal seaweed extract supplementation on suckling piglet growth, humoral immunity, selected microflora, and immune response after an ex vivo lipopolysaccharide challenge. J Anim Sci. (2012) 90:505–14. 10.2527/jas.2010-3243 21948611

[20] Heim G Sweeney T O'Shea CJ Doyle DN O'Doherty JV. Effect of maternal supplementation with seaweed extracts on growth performance and aspects of gastrointestinal health of newly weaned piglets after challenge with enterotoxigenic *Escherichia coli* K88. Br J Nutr. (2014) 112:1955–65. 10.1017/S0007114514003171 25345748

[21] Bouwhuis MA Sweeney T McDonnell MJ Doyle DN Thornton K. The effect of maternal and postweaning seaweed extract supplementation on gut health in pigs after weaning and response to enterotoxigenic *Escherichia coli* K88 challenge. J Anim Sci. (2016) 94:395–8. 10.2527/jas.2015-9870

[22] Han X Piao XS Zhang HY Li PF Yi JQ Zhang Q. Forsythia suspensa extract has the potential to substitute antibiotic in broiler chicken. Asian Austral J Anim Sci. (2012) 25:569–76. 10.5713/ajas.2011.11425 25049598 PMC4092897

[23] Long SF Liu L Liu SJ Mahfuz S Piao XS. Effects of *Forsythia suspense* extract as an antibiotics substitute on growth performance, nutrient digestibility, serum antioxidant capacity, fecal *Escherichia coli* concentration and intestinal morphology of weaned piglets. Animals. (2019) 9:729. 10.3390/ani9100729 31561574 PMC6826561

[24] Long SF Wu D He TF Piao XS. Dietary supplementation with *Forsythia suspensa* extract during late gestation improves reproductive performance, colostrum composition, antioxidant status, immunoglobulin, and inflammatory cytokines in sows and newborn piglets. Anim Feed Sci Technol. (2020) 271:114700. 10.1016/j.anifeedsci.2020.114700

[25] NRC. Nutrient Requirements of Swine. 11th revision edition. Washington, DC: National Academy Press (2012).

[26] Xu Y Zeng Z Xu X Tian Q Ma X Long S. Effects of the standardized ileal digestible valine: lysine ratio on performance, milk composition and plasma indices of lactating sows. Anim Sci J. (2016) 88:1082–92. 10.1111/asj.12753 27921350

[27] AOAC. Official Method of Analysis. 15th edition. Washington, DC: AOAC (1990).

[28] AOAC. Official Methods of Analysis, 19th edition. Arlington, VA: AOAC (2012).

[29] Hu P Zhao F Zhu W Wang J. Effects of early-life lactoferrin intervention on growth performance, small intestinal function and gut microbiota in suckling piglets. Food Funct. (2019) 10:5361–73. 10.1039/C9FO00676A 31393487

[30] Zhang LH Li M Shang QH Hu JX Long SF Piao XS. Effects of maternal 25-hydroxycholecalciferol on nutrient digestibility, milk composition and fatty-acid profile of lactating sows and gut bacterial metabolites in the hindgut of suckling piglets. Arch Anim Nutr. (2019) 73:271–86. 10.1080/1745039X.2019.1620041 31192703

[31] Long SF Xu YT Pan L Wang QQ Wang CL Wu JY. Mixed organic acids as antibiotic substitutes improve performance, serum immunity, intestinal morphology and microbiota for weaned piglets. Anim Feed Sci Technol. (2018) 235:23–32. 10.1016/j.anifeedsci.2017.08.018

[32] Cole JR Wang Q Fish JA Chai B Tiedje JM. Ribosomal database project: tools and data for high throughput rRNA analysis. Nucleic Acids Res. (2013) 42:633–42. 10.1093/nar/gkt1244 24288368 PMC3965039

[33] Mahnert A Moissl-Eichinger C Berg G. Microbiome interplay: plants alter microbial abundance and diversity within the built environment. Front. Microbiol. (2015) 6:887. 10.3389/fmicb.2015.00887 26379656 PMC4552223

[34] Di J Shengguo Z Pengpeng W Nan Z Dengpan B Yves B. Insights into abundant rumen ureolytic bacterial community using rumen simulation system. Front Microbiol. (2016) 7:887. 10.3389/fmicb.2016.01006 27446045 PMC4923134

[35] Segata N Izard J Waldron L Gevers D Miropolsky L Garrett WS. Metagenomic biomarker discovery and explanation. Genome Biol. (2011) 12:60. 10.1186/gb-2011-12-6-r60 21702898 PMC3218848

[36] SAS. SAS/STAT Users Guide: Statistics, Version 9.2 Ed. Cary, NC: SAS Inc (2008).

[37] Piao XL Jang MH Cui J Piao XS. Lignans from the fruits of *Forsythia suspensa*. Bioorg Med Chem Lett. (2008) 18:1980–4. 10.1016/j.bmcl.2008.01.115 18295482

[38] Lambert JD Elias RJ. The antioxidant and pro-oxidant activities of green tea polyphenols: a role in cancer prevention. Arch Biochem Biophys. (2010) 501:65–72. 10.1016/j.abb.2010.06.013 20558130 PMC2946098

[39] Wu PF Zhang Z Wang F Chen JG. Natural compounds from traditional medicinal herbs in the treatment of cerebral ischemia/reperfusion injury. Acta Pharmacol Sin. (2010) 31:1523–31. 10.1038/aps.2010.186 21127495 PMC4002952

[40] Sordillo LM Contreras GA Aitken SL. Metabolic factors affecting the inflammatory response of periparturient dairy cows. Anim Health Res Rev. (2009) 10:53–63. 10.1017/S1466252309990016 19558749

[41] Long SF Piao XS. Effects of dietary *Forsythia suspensa* extract supplementation to lactating sows and nursery pigs on post-weaning performance, antioxidant capacity, nutrient digestibility, immunoglobulins and intestinal health. J Anim Sci. (2021) 99:skab142. 10.1093/jas/skab142 34014312 PMC8372046

[42] Valko M Rhodes CJ Moncol J Izakovic M Mazur M. Free radicals, metals and antioxidants in oxidative stress-induced cancer. Chem Biol Interact. (2006) 160:1–40. 10.1016/j.cbi.2005.12.009 16430879

[43] Huang C Lin Y Su H Ye D. Forsythiaside protects against hydrogen peroxide-induced oxidative stress and apoptosis in PC12 cell. Neuroche Res. (2015) 40:27–35. 10.1007/s11064-014-1461-5 25344274

[44] Meng Q Guo T Li G Sun S He S Cheng B. Dietary resveratrol improves antioxidant status of sows and piglets and regulates antioxidant gene expression in placenta by Keap1-Nrf2 pathway and Sirt1. J Anim Sci Biotechnol. (2018) 9:34. 10.1186/s40104-018-0248-y 29713468 PMC5909222

[45] Li B Nasser MI Masood M Adlat S Huang Y Yang B. Efficiency of traditional Chinese medicine targeting the Nrf2/HO-1 signaling pathway. Biomed Pharmacother. (2020) 126:110074. 10.1016/j.biopha.2020.110074 32163746

[46] Harder B Jiang T Wu T Tao S De La Vega MR Tian W. Molecular mechanisms of Nrf2 regulation and how these influence chemical modulation for disease intervention. Biochem Soc Trans. (2015) 43:680–6. 10.1042/BST20150020 26551712 PMC4613518

[47] Li X Ye F Li L Chang W Wu X Chen J. The role of HO-1 in protection against lead-induced neurotoxicity. Neurotoxicology. (2016) 52:1–11. 10.1016/j.neuro.2015.10.015 26542248

[48] Che M Wang R Li X Wang HY Zheng XS. Expanding roles of superoxide dismutases in cell regulation and cancer. Drug Discov Today. (2016) 21:143–9. 10.1016/j.drudis.2015.10.001 26475962 PMC4724522

[49] Xiong Y Shie FS Zhang J Lee CP Ho YS. The protective role of cellular glutathione peroxidase against trauma-induced mitochondrial dysfunction in the mouse brain. J. Stroke Cerebrovasc. (2004) 13:129–37. 10.1016/j.jstrokecerebrovasdis.2004.05.001 17903964

[50] Lei XG. Glutathione peroxidase-1 gene knockout on body antioxidant defense in mice. Biofactors. (2001) 14:93–9. 10.1002/biof.5520140113 11568445

[51] Loboda A Damulewicz M Pyza E Jozkowicz A Dulak J. Role of Nrf2/HO-1 system in development, oxidative stress response and diseases: an evolutionarily conserved mechanism. Cell Mol Life Sci. (2016) 73:3221–47. 10.1007/s00018-016-2223-0 27100828 PMC4967105

[52] Wang Y Zhao H Lin C Ren J Zhang S. Forsythiaside A exhibits anti-inflammatory effects in LPS-stimulated BV2 microglia cells through activation of Nrf2/HO-1 signaling pathway. Neurochem. Res. (2016) 41:659–65. 10.1007/s11064-015-1731-x 26498935

[53] Jiang WL Zhang SP Zhu HB. Forsythoside B protects against experimental sepsis by modulating inflammatory factors. Phytoth Res. (2012) 26:981–7. 10.1002/ptr.3668 22147417

[54] Jia J Zhang F Li Z Qin X Zhang L. Comparison of fruits of forsythia suspensa at two different maturation stages by NMR-based metabolomics. Molecules. (2015) 20:10065–81. 10.3390/molecules200610065 26035103 PMC6272181

[55] Cheng G Zhao Y Li H Wu Y Li X Han Q. Forsythiaside attenuates lipopolysaccharide-induced inflammatory responses in the bursa of fabricius of chickens by downregulating the NF-κB signaling pathway. Exp Ther Med. (2014) 7:179–84. 10.3892/etm.2013.1378 24348786 PMC3861409

[56] Liu H Zhang J Zhang S Yang F Thacker PA Zhang G. Oral administration of *Lactobacillus fermentum* I5007 favors intestinal development and alters the intestinal microbiota in formula-fed piglets. J Agri Food Chem. (2014) 62:860–6. 10.1021/jf403288r 24404892

[57] Feldman GJ Mullin JM Ryan MP. Occludin: structure, function and regulation. Adv Drug Deliver Rev. (2005) 57:883–917. 10.1016/j.addr.2005.01.009 15820558

[58] Chen L Li L Han Y Lv B Zou S Yu Q. Tong-fu-li-fei decoction exerts a protective effect on intestinal barrier of sepsis in rats through upregulating ZO-1/occludin/claudin-1 expression. J Pharmacol Sci. (2020) 143:89–96. 10.1016/j.jphs.2020.02.009 32173265

[59] Feng W Ao H Peng C. Gut microbiota, short-chain fatty acids, and herbal medicines. Front Pharmacol. (2018) 9:1354. 10.3389/fphar.2018.01354 30532706 PMC6265305

[60] Liu WT Mino T Nakamura K Matsuo T. Role of glycogen in acetate uptake and polyhydroxyalkanoate synthesis in anaerobic-aerobic activated sludge with a minimized polyphosphate content. J Ferment Bioeng. (1994) 77:535–40. 10.1016/0922-338X(94)90124-4

[61] Bergman E N. Energy contributions of volatile fatty acids from the gastrointestinal tract in various species. Physiol Rev. (1990) 70:567–90. 10.1152/physrev.1990.70.2.567 2181501

[62] Partanen K H Mroz Z. Organic acids for performance enhancement in pig diets. Nutr Res Rev. (1999) 12:117–45. 10.1079/095442299108728884 19087448

[63] Yin J Li Y Han H Chen S Gao J Liu G. Melatonin reprogramming of gut microbiota improves lipid dysmetabolism in high-fat diet-fed mice. J Pineal Res. (2018) 65:12524. 10.1111/jpi.12524 30230594

[64] Zhang S Zhong G Shao D Wang Q Hu Y Wu TX. Dietary supplementation with *Bacillus subtilis* promotes growth performance of broilers by altering the dominant microbial community. Poultry Sci. (2021) 100:100935. 10.1016/j.psj.2020.12.032 33652528 PMC7936199

[65] Shi SR Wu S Shen YR Zhang S Xiao YQ He X. Iron oxide nanozyme suppresses intracellular *Salmonella enteritidis* growth and alleviates infection in vivo. Theranostics. (2018) 8:6149–62. 10.7150/thno.29303 30613289 PMC6299686

[66] Xiao YQ Zhang S Tong HB Shi SR. Comprehensive evaluation of the role of soy and isoflavone supplementation in humans and animals over the past two decades. Phytother Res. (2018) 32:384–94. 10.1002/ptr.5966 29193539

[67] Yang Q Huang X Zhao S Sun W Yan Z Wang P. Structure and function of the fecal microbiota in diarrheic neonatal piglets. Front Microbiol. (2017) 8:502. 10.3389/fmicb.2017.00502 28392784 PMC5364137

[68] Mroz Z Jongbloed AW Partanen KH Vreman K Kemme PA Kogut J. The effects of calcium benzoate in diets with or without organic acids on dietary buffering capacity, apparent digestibility, retention of nutrients, and manure characteristics in swine. J Anim Sci. (2000) 78:2622–32. 10.2527/2000.78102622x 11048928

[69] Liu R Lai K Xiao Y Ren J. Comparative pharmacokinetics of chlorogenic acid in beagles after oral administrations of single compound, the extracts of *Lonicera japanica*, and the mixture of chlorogenic acid, baicalin, and *Forsythia suspense*. Pharm Biol. (2017) 55:1234–8. 10.1080/13880209.2017.1296002 28260397 PMC6130634

[70] Jiao J Gai QY Fu YJ Zu YG Luo M Wang W. Application of white-rot fungi treated fructus forsythiae shell residue as a low-cost biosorbent to enrich forsythiaside and phillygenin. Chem Eng Sci. (2012) 74:244–55. 10.1016/j.ces.2012.02.057

[71] Tamanai-Shacoori Z Smida I Bousarghin L Loreal O Meuric V Fong SB. *Roseburia* spp.: a marker of health? Future Microbiol. (2017) 12:157–70. 10.2217/fmb-2016-0130 28139139

[72] Guilloteau P Martin L Eeckhaut V Ducatelle R Zabielski R Van Immerseel F. From the gut to the peripheral tissues: the multiple effects of butyrate. Nutr Res Rev. (2010) 23:366–84. 10.1017/S0954422410000247 20937167

[73] Chen J Mao Y Xing C Hu R Xu Z Cao H. Traditional Chinese medicine prescriptions decrease diarrhea rate by relieving colonic inflammation and ameliorating caecum microbiota in piglets. Evid Based Complement Alternat Med. (2020) 2020:3647525. 10.1155/2020/3647525 32351595 PMC7178461

