# Supplementary material for: Maternal Dietary Forsythia suspensa Extract Supplementation Induces Changes in Offspring Antioxidant Status, Inflammatory Responses, Intestinal Development, and Microbial Community of Sows
Source: Front Vet Sci. 2022 Jul 15;9:926822. doi: 10.3389/fvets.2022.926822 (PMC9334818; doi:10.3389/fvets.2022.926822)
Supplement: Supplementary file 1 [file Table_1.pdf]

SUPPLEMENTED TABLE | 1. The primer sequences for qRT-PCR of antioxidant relative mRNA expression in liver of suckling pigs

Gene	Primer sequence (5'-3')	Size (bp)	GenBank accession number
GAPDH	Forward: CTGCCGCCTGGAGAAACCT<br>Reverse: GCTGTAGCCAAATTCATTGTCG	226	NM_001206359.1
SOD1	Forward: GAGACCTGGGCAATGTGACTG<br>Reverse: GCCAAACGACTTCCAGCAT	190	NM_001190422.1
GPx1	Forward: CAGGCGGCGGGTTCG<br>Reverse: TGAGGGCAGTGGCATCGT	129	NM_214201.1
Nrf2	Forward: ACCTGTGCCTGCTGGATTGAGA<br>Reverse: GGTTCGTTACCACTGAGCCATAGC	162	XM_005671981.3
HO-1	Forward: TGGTCTCTTGACTGGCTTCCTTGT<br>Reverse: GCTTCTGGCTGGCTCCATTCTC	245	NM_001004027.1

Note: SOD1: superoxide dismutase 1; GPx1, glutathione peroxidase 1; Nrf2, nuclear factor E2-related factor 2; HO-1: heme oxygenase 1.
